# Altered hippocampus synaptic function in selenoprotein P deficient mice

**DOI:** 10.1186/1750-1326-1-12

**Published:** 2006-09-19

**Authors:** Melinda M Peters, Kristina E Hill, Raymond F Burk, Edwin J Weeber

**Affiliations:** 1 Department of Molecular Physiology and Biophysics, Vanderbilt University, Nashville, USA; 2 Department of Medicine, Vanderbilt University, Nashville, USA; 3 Department of Pathology, Vanderbilt University, Nashville, USA; 4 Department of Pharmacology, Vanderbilt University, Nashville, USA; 5 Vanderbilt Kennedy Center for Research on Human Development, Vanderbilt University Medical Center, Nashville, USA

## Abstract

Selenium is an essential micronutrient that function through selenoproteins. Selenium deficiency results in lower concentrations of selenium and selenoproteins. The brain maintains it's selenium better than other tissues under low-selenium conditions. Recently, the selenium-containing protein selenoprotein P (Sepp) has been identified as a possible transporter of selenium. The targeted disruption of the selenoprotein P gene (*Sepp1*) results in decreased brain selenium concentration and neurological dysfunction, unless selenium intake is excessive However, the effect of selenoprotein P deficiency on the processes of memory formation and synaptic plasticity is unknown. In the present studies *Sepp1*(-/-) mice and wild type littermate controls (*Sepp1*(+/+)) fed a high-selenium diet (1 mg Se/kg) were used to characterize activity, motor coordination, and anxiety as well as hippocampus-dependent learning and memory. Normal associative learning, but disrupted spatial learning was observed in *Sepp1*(-/-) mice. In addition, severe alterations were observed in synaptic transmission, short-term plasticity and long-term potentiation in hippocampus area CA1 synapses of *Sepp1*(-/-) mice on a 1 mg Se/kg diet and *Sepp1*(+/+) mice fed a selenium-deficient (0 mg Se/kg) diet. Taken together, these data suggest that selenoprotein P is required for normal synaptic function, either through presence of the protein or delivery of required selenium to the CNS.

## Background

Selenium (Se) is a naturally occurring micronutrient that is essential for several known major metabolic pathways, including; thyroid hormone metabolism [1-3] and antioxidant defense systems [4,5] in both humans and rodents. Dietary selenium can exist as selenomethionine, selenocysteine, selenate or selenite [6] and is incorporated as selenocysteine into a subset of specific selenium-dependent proteins (selenoproteins) [7]. Of particular interest are the selenoproteins involved in oxidative stress, such as the glutathione peroxidase enzymes (classical GPX-1, gastrointestinal GPX-2, plasma GPX-3, phospholipid hydroperoxide GPX-4) and the thioredoxin reductase 1 and 2 (TR) [8,9]. The dietary intake of selenium has a delicate balance between the harmful effects of excessive selenium uptake leading to selenium toxicity and the damaging effects on selenoprotein function during selenium deficiency. Interestingly, the CNS appears to be resistant to fluctuations in selenium and can maintain stable levels despite near complete depletion of dietary selenium uptake [10]. This suggests that the process of selenium transport needed for selenocysteine-protein incorporation is important for normal CNS function.

Approximately 60% of selenium in plasma is present as selenoprotein P [11]. This protein differs from other selenoproteins in that it incorporates up to 10 Se atoms per molecule in the form of selenocysteine as opposed to the single selenocysteine incorporated in other selenoproteins [12]. Selenoprotein P is abundant throughout the body suggesting that one function is to serve as a primary transporter in systemic selenium delivery [13,14]. This is especially evident in the CNS where selenoprotein P levels can be maintained independent of plasma selenium [15]. However, genetic ablation of selenoprotein P results in reduced, but not a commensurate decrease in CNS-associated selenium levels, suggesting that other selenium proteins compensate for the selenoprotein P deficiency and supporting the hypothesis that basal selenium levels are essential for the brain and have a priority for available selenium [3,16]. *Sepp1*(-/-) mice fed a selenium-deficient diet show severe motor dysfunction associated with neuron degeneration, which can be prevented by supplementation of high dietary selenium [16-18].

Reduced dietary selenium can have significant effects on levels of selenoproteins involved in oxidative stress, such as glutathione peroxidases, thioredoxin reductases and methionine sulfoxide reductases [19,20]. Selenium, through the incorporation into selenoproteins, provides protection from reactive oxygen species (ROS)-induced cell damage [21]. Because oxidative stress, and subsequent production of ROS, has been implicated in neurodegenerative disorders, such as Alzheimer's disease, Parkinson's disease and Duchenne muscular dystrophy [22], there may also be a role for selenium in these disorders. In the present study we examined the consequences of selenoprotein P deficiency on cognitive capacity and synaptic function with a focus on the hippocampus, an area of the CNS intimately involved in learning and memory processes. *Sepp1*(-/-) mice demonstrate no overt behavior phenotype, but were found to have a subtle disruption in acquisition of spatial learning and memory. In contrast, synaptic transmission is altered and short- and long-term synaptic plasticity is severely disrupted in area CA1 of hippocampus. Interestingly, we found that when *Sepp1*(+/+) mice were fed a low Selenium diet (0 mg/kg), they too exhibited altered synaptic transmission and synaptic plasticity. Our observations suggest an important role for both selenoprotein P and dietary selenium in overall proper synaptic function.

## Materials and methods

### Animals

Littermates obtained from *Sepp1*(+/-) crosses were genotyped as described previously [16]. This line has been back-crossed to C57BL6/J ten times. Animals were fed a Torula yeast-based diet supplemented with either 0 mg Se/kg or 1 mg Se/kg in the form of sodium selenite [18] and housed in a 10/14 hr light/dark cycle. Mice used for behavior were fed a diet of 1 mg Se/kg. Behavior testing was performed with 3–5 month old males and females during the light cycle. No gender-dependent differences were observed in any of the tests, therefore data were combined for graphical presentation and statistical analysis. All animal testing procedures were approved by the Institutional Animal Care and Use Committee of Vanderbilt University and followed the NIH guidelines for the care and use of laboratory animals.

### Histology

*Sepp1*(-/-) and *Sepp1*(+/+) mice fed 1 mg Se/kg and *Sepp1*(+/+) mice fed 0 mg Se/kg were anesthetized by isofluorane and transcardially perfused with ice-cold clearing solution (0.1 M phosphate-buffered saline solution), followed by 4% paraformaldehyde (PFA) in 0.1 M phosphate buffer. Brains were then dissected, incubated overnight in 4% PFA at 4°C and cryoprotected with 30% sucrose. 40 μm coronal sections were cut using a cryostat (Leica CM 3050S) and mounted on gelatin-coated slides. Tissue was stained with 0.1% toludine blue to visualize the cellular structure of the hippocampus.

### Rotorod

Starting speed for the rod began at 4 rpm and increased to 40 rpm over a 5 min period. Latency to fall was recorded as the time in which the mouse fell off the rod or failed to continue walking on the rod after two revolutions. The mice were given four trials per day with an inter-trial interval of 1 hour for two consecutive days. Data represent the mean ± SEM. Student's *t *test was used for statistical analysis with p < 0.05 as significance criteria.

### Open field

Total distance traveled in the open field chamber (27 × 27 cm) during 15 min under standard room lighting conditions was assayed. Mouse activity was measured by 16 photoreceptor beams on all sides of the chamber connected to an Activity Monitor program (Med Associates, Inc). Zone analysis was performed to determine the time spent in the center of the chamber (designated by an 8 cm × 8 cm region) compared to the perimeter. Data represent the mean ± SEM. Student's *t *test was used for statistical analysis with p < 0.05 as significance criteria.

### Elevated plus maze

The elevated plus maze apparatus consisted of two opposing open arms (30 cm × 5 cm) and two opposing closed arms (30 cm × 5 cm × 15 cm) connected by a central square platform and positioned 40 cm above the ground. Mice were placed in the open arms facing the closed arms at the beginning of the 5 min session. Data represent the total time spent in the various locations ± SEM. Student's *t *test was used for statistical analysis with p < 0.05 as significance criteria.

### Conditioned fear

Training for contextual and cued fear conditioning consisted of a 2 min exploration period, followed by two conditioned stimulus (CS)-unconditioned stimulus (US) pairings separated by 90 sec (tone: 85 dB white noise, 30 s duration; foot shock intensity: 0.5 mA, 2 s duration). Context tests were performed in the conditioning chamber 2 hrs, 24 hrs, and 1 week post training. Cue tests were performed in a dissimilar chamber 2 and 24 hours post training; baseline freezing was monitored before presentation of the tone (85 dB white noise, 3 min duration). Freezing was defined as the absence of movement for 2 sec and measured objectively by a motion monitoring program (Med Associates, Inc). Data represent the mean ± SEM. Student's *t *test was used for statistical analysis with *p *< 0.05 as significance criteria.

### Water maze

Training for the hidden platform version of the Morris water maze consisted of four trials (60 s maximum; inter-trial interval, 60 min) each day with the starting location changing for each trial to avoid quadrant bias. On days 5, 7 and 10, a probe trial was administered before training by removing the platform and monitoring the animals' trajectory over 60 sec. The visible platform test was performed 2 hours after the last training session on day 10. A new platform was placed in the quadrant opposite from where they were trained. Above the submerged platform was a large red flag. The mice were placed in the opposite quadrant and latencies to find the new platform were recorded. Animal swim paths were objectively recorded with a video tracking system (HVS Image Analyzing VP-200). Data represent the mean ± SEM. Student's *t *test linear regression and χ^2 ^tests were used for statistical analysis with *p *< 0.05 as significance criteria.

### Electrophysiology

400 μm Hippocampus slices were prepared with a vibratome from 3–5 month old mice as described previously [23]. Briefly, animals were sacrificed and brains were quickly placed in ice cold high sucrose cutting solution (in mM: 110 sucrose, 6 NaCl, 3 KCl, 26 NaHCO_3_, 1.25 NaH_2_PO_4_, 7 MgCl_2_, 0.5 CaCl_2_, 0.6 sodium ascorbate and 10 glucose, pH 7.3–7.4) and oxygenated with 95% O_2_/5% CO_2_. The hippocampus was dissected and allowed to equilibrate at RT in a 1:1 solution of cutting solution and artificial cerebrospinal fluid (ACSF: containing in mM: 125 NaCl, 2.5 KCl, 1.25 NaH_2_PO_4_, 26 NaHCO_3_, 1.2 MgCl_2_, 2.0 CaCl_2_, and 10 glucose, pH 7.3–7.4) before recovering in oxygenated ACSF at 30°C in the interface recording chamber. All slices were permitted a minimum 1 hr recovery time before synaptic recording. Standard techniques as reported previously [24] were used to obtain extracellular field recordings and delivery of paired pulse and 100 Hz stimulations. Due to the high mortality of *Sepp1*(-/-) fed a selenium-deficient diet [18], *Sepp1*(-/-) mice used for electrophysiology were maintained on the high (1 mg Se/kg) selenium diet.

## Results

### Normal hippocampal morphology in *Sepp1*(-/-) mice

**Figure 1:**
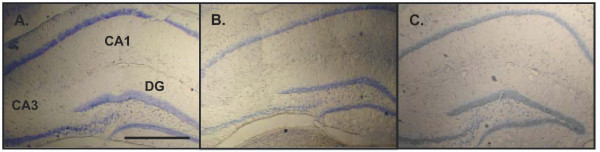
**Normal hippocampal morphology in *Sepp1*(-/-) mice**. 0.1% Toludine blue staining of the cell bodies in 40 μm hippocampal slices obtained from **(A) ***Sepp1*(-/-) mice fed 1 mg Se/kg **(B) ***Sepp1*(+/+) mice fed 1 mg Se/kg and **(C) ***Sepp1*(+/+) mice fed 0 mg Se/kg. The major regions of the hippocampus, dentate gyrus (DG), CA3 and CA1, are indicated in panel A. Magnification at 4×. Scale bar roughly 1 mm.

The generation and metabolic characterization of the *Sepp1*(-/-) mice has been described previously [16]. The following experiments focus on hippocampus-dependent learning and memory, as well as direct measurement of synaptic transmission and plasticity in area CA1 of the hippocampus. Therefore we performed a gross morphologic examination of the CNS in general, and hippocampus specifically, in order to identify any potential differences due to developmental abnormalities. We find that *Sepp1*(+/+) and *Sepp1*(-/-) fed 1 mg Se/kg and *Sepp1*(+/+) fed a 0 mg Se/kg diet does not alter the gross morphology of the CNS in general (data not shown) or the size and organization of the hippocampus (Figure 1). In addition, chronic depletion of selenium from the diet of adolescent mice does not result in gross morphological changes in the hippocampus. These observations also suggest that any hippocampus-dependent behavioral or electrophysiological phenotypes we observe are not likely the result of gross morphological abnormalities in the hippocampus, but rather altered Sepp- or selenium-dependent metabolic or signaling processes.

### *Sepp1*(-/-) mice show motor coordination defects but normal levels of locomotor activity and anxiety

**Figure 2:**
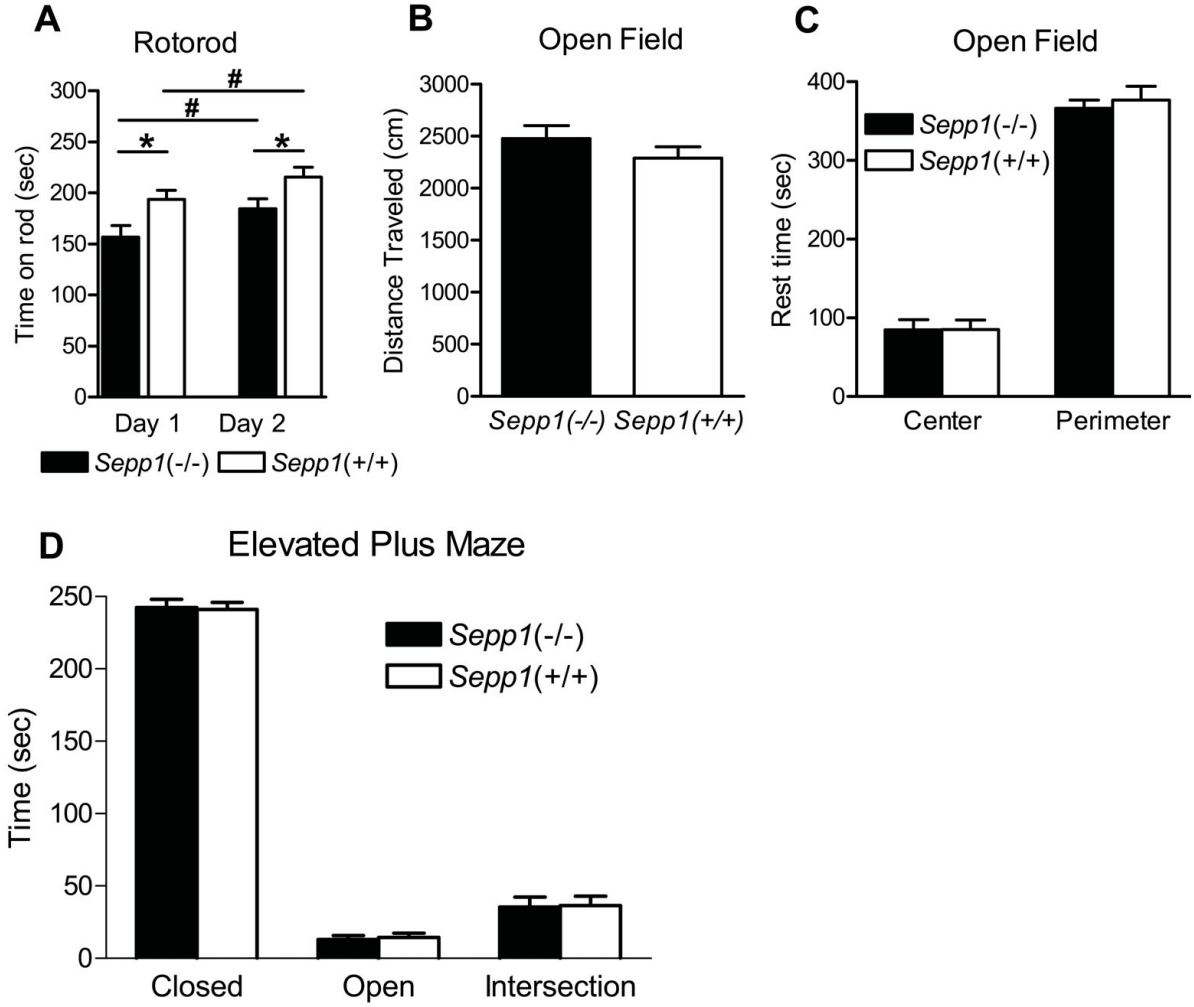
***Sepp1*(-/-) mice show reduced motor coordination and normal anxiety**. **(A) **Average latency to fall off the rotorod from 4 trials for 2 consecutive days (n = 16 mice per genotype). Statistical analysis of Day 1 *Sepp1*(-/-):*Sepp1*(+/+) *p < 0.0001, Day 2 *Sepp1*(-/-):*Sepp1*(+/+) *p = 0.0008, Day 1 *Sepp1*(-/-):Day 2 *Sepp1*(-/-) #p = 0.006, Day 1 *Sepp1*(+/+):Day 2 *Sepp1*(+/+) #p = 0.01. **(B) **Total distance traveled during 15 min in the open field (n = 16 mice per genotype, p = 0.26). **(C) **Zone analysis of the time spent in the center of the open field during 15 min. **(D) **Time spent resting in each of the arm conditions of the elevated plus maze (n = 16 mice per genotype).

Because it has been shown that selenoprotein P plays a role in maintenance of selenium levels in the brain and that it may be linked to certain neurodegenerative disorders, we examined potential *Sepp1*(-/-) behavioral phenotypes in several well-characterized behavioral paradigms. These tests also serve as necessary controls when elucidating the role of selenoprotein P in cognitive processes. Both Hill et al (2004) and Schweizer et al (2004) performed rotorod experiments to test overall locomotor function in *Sepp1*-deficient mice and found reduced motor coordination [18,22]. We performed a modified version of their protocol to include motor learning over time. For both days of training, we observed that *Sepp1*(-/-) mice had shorter latencies to fall off the accelerating rotorod than their littermate controls (Figure 2A). We also found a statistically significant increase in latencies over the two day period for both genotypes. In agreement with previous reports, we found motor coordination is compromised in *Sepp1*(-/-) mice and we extend those findings to include that motor learning is preserved in the absence of selenoprotein P.

Previous research of selenium deficiency in mice revealed reduced open field activity and that the mice spent less time in the center of the field indicating increased anxiety-like behavior [25]. Thus, we performed the same open field test to assay general locomotor activity and anxiety. In comparing the total distance traveled during a 15 min exposure to the chamber we found no statistical difference between the *Sepp1*(-/-) mice and *Sepp1*(+/+) (Figure 2B). Zone analysis revealed that both genotypes spent equivalent amounts of time in the center of the chamber and the greatest amount of time in the surrounding areas, nearest to the walls of the chamber (Figure 2C). These results indicate that, unlike dietary selenium deficiency, the *Sepp1*(-/-) mice have normal exploratory tendencies and behave similar to their littermate controls when introduced to a novel open field.

A more sensitive analysis of anxiety can be achieved with the elevated plus maze. Both genotypes demonstrated a similar pattern of behavior when presented with the elevated plus maze. Equivalent amounts of time were spent in the open arms of the maze with the preponderance of time spent in the closed arms (Figure 2D). These observations are consistent with the open field analysis and suggest that genetic elimination of selenoprotein P does not have an adverse effect on overall anxiety-like behavior.

### *Sepp1*(-/-) mice exhibit normal associative fear learning and impaired spatial learning

**Figure 3:**
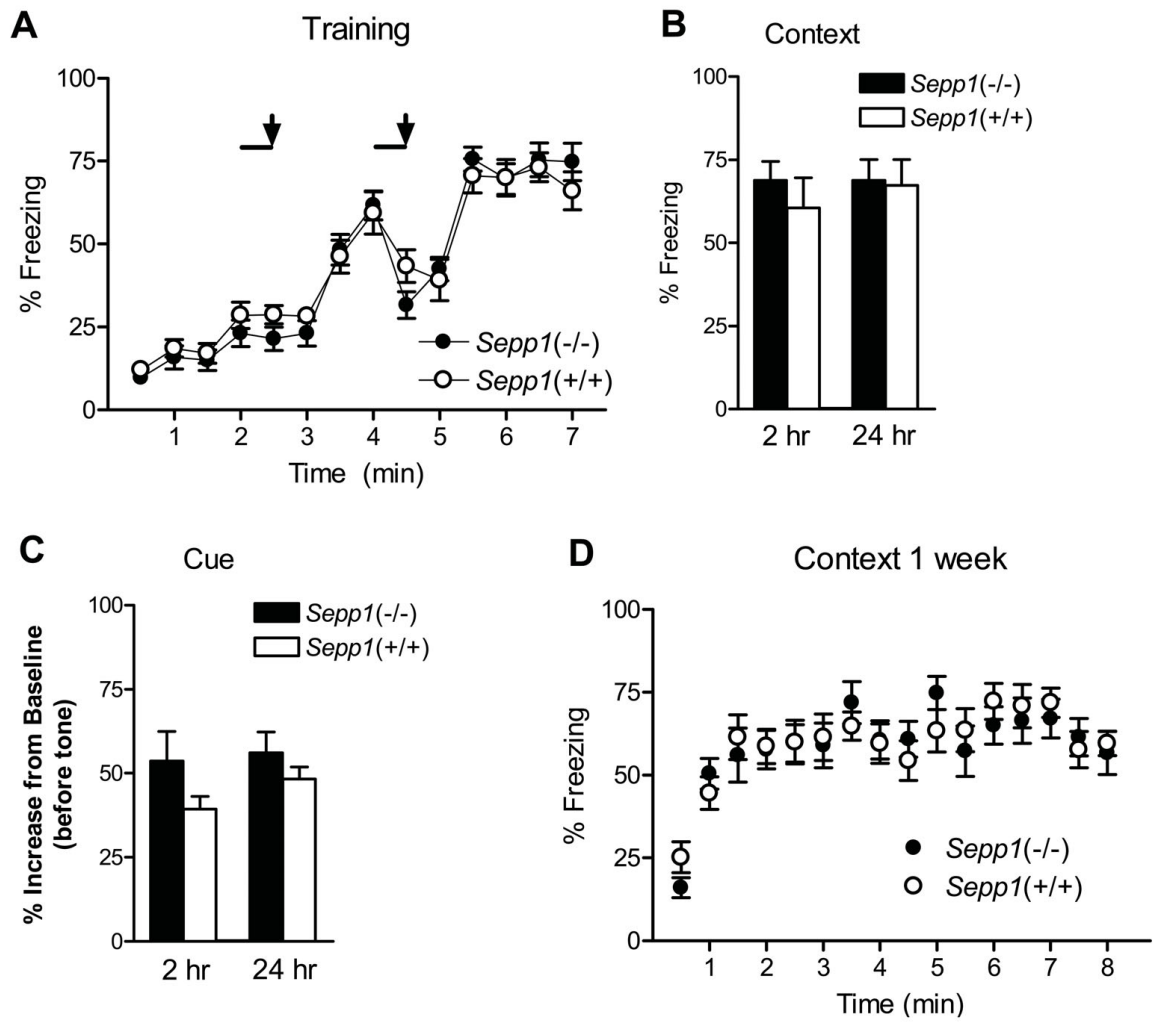
***Sepp1*(-/-) mice exhibit normal associative conditioned fear learning and memory**. **(A) **Freezing during the two trial, paired conditioned stimulus (CS: 85dB tone represented by black line) and unconditioned stimulus (US: 0.5mA foot shock represented by the arrow) training paradigm. Both genotypes respond comparably to training (n = 16 mice per genotype). **(B) **Freezing during the context tests (2.5 min) performed 2 hrs and 24 hrs post training. Both genotypes learned to associate the context with the US equally well. **(C) **Freezing during the cued tests performed 2 hrs and 24 hrs post training. Both genotypes learned to associate the CS with the US equally well. Graph shows the % increase in freezing to the CS compared to baseline freezing before presentation of the CS. **(D) **Freezing during the context test (8 min) performed 1 week post training. Both genotypes exhibited long-term memory for association of the context with the US.

To measure associative fear learning and memory in *Sepp1*(-/-) mice, we used an aversive stimulus (unconditioned stimulus-US, mild foot shock) paired with an auditory cue (conditioned stimulus-CS, 84 dB tone). During training, mice showed similar freezing rates when presented with the two CS-US pairings over the 7 min period (Figure 3A). To assess short-term memory retrieval, mice were subjected to context and cue tests 2 hrs post training. No difference was observed between the genotypes in either short-term memory tests (Figure 3B and 3C). Long-term associative memory formation was assayed 24 hrs after training. Again the *Sepp1*(-/-) mice were indistinguishable from the *Sepp1*(+/+) in both the context and cue tests (Figure 3B and 3C). Finally, to assess hippocampus-dependent long-term memory retention, we placed the mice back in the training context 1 week post training for 8 min. No difference in freezing while in the context was observed between the *Sepp1*(-/-) and *Sepp1*(+/+) mice (Figure 3D). Taken together, these results suggest that selenoprotein P is not essential for contextual or cued associative learning and memory.

**Figure 4:**
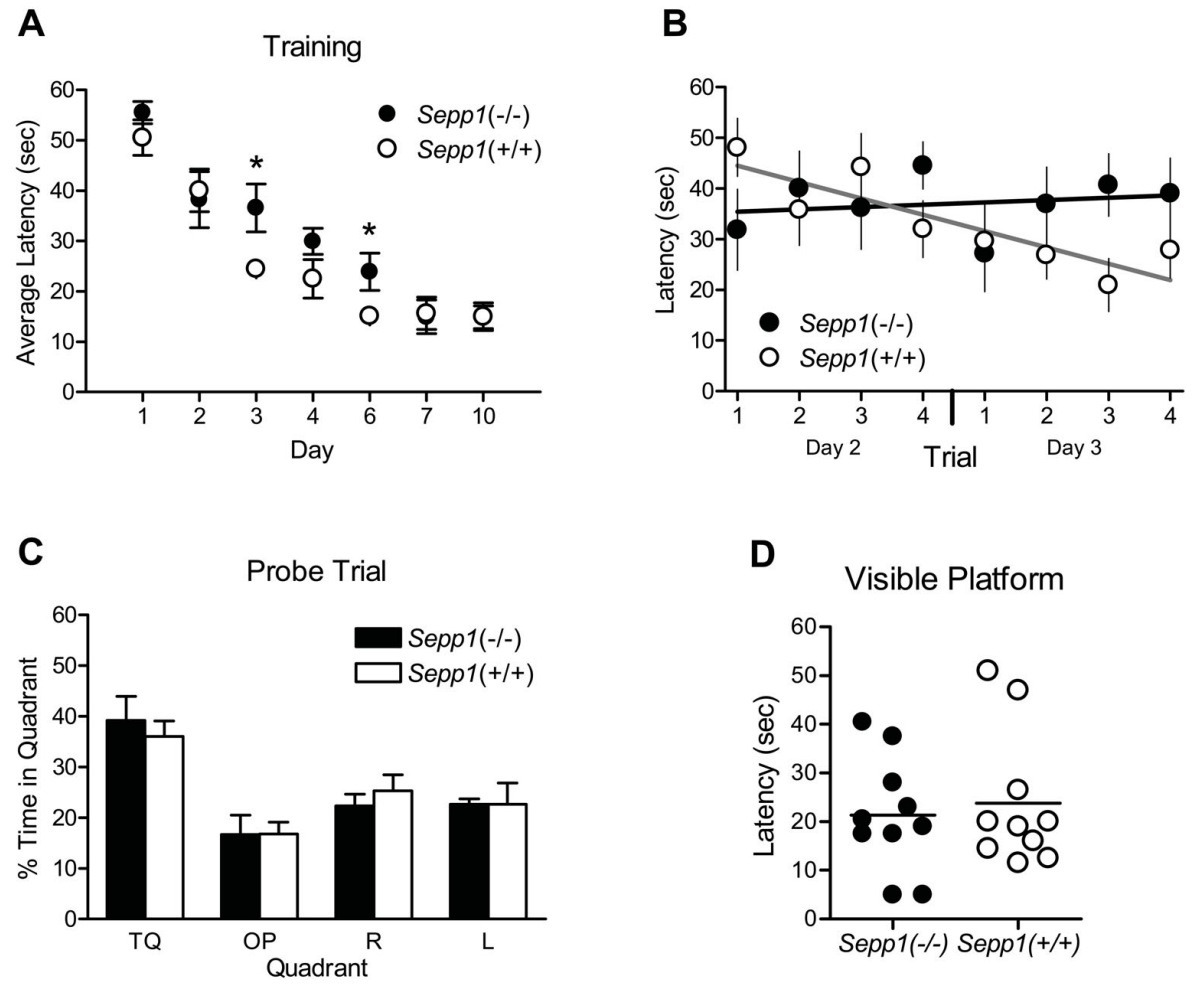
***Sepp1*(-/-) mice exhibit impaired acquisition of spatial learning and memory**. **(A) **Average escape latency from 4 trials per day. *Sepp1*(-/-) mice showed a significantly longer latency on days 3 and 6 (n = 10 mice per genotype, Day 3 *p = 0.04, Day 6 *p = 0.04). **(B) **Linear regression of escape latencies per trial from days 2 to 3 (*Sepp1*(-/-) slope = 0.5, *Sepp1*(+/+) slope = -3.5). **(C) **Time spent searching the 4 quadrants for the hidden platform during a 60 sec probe trial on day 5 (TQ-target quadrant, OP-opposite, R-right, L-left). Both genotypes selectively searched the target quadrant. **(D) **Day 10 latencies to locate a visible platform in the opposite quadrant from training. Both genotypes demonstrated similar ability to locate the visible platform.

The disruption of spatial learning during selenium deficiency has been previously reported [25]. Despite the lack of a phenotype in our associative fear conditioning paradigm, we performed a standard spatial learning test using the Morris hidden platform water maze paradigm. For this study, both *Sepp1*(-/-) and *Sepp1*(+/+) mice were trained to locate a platform submerged in a circular pool filled with opaque water. Distal visual cues were positioned outside the pool and the latency to escape via the platform was recorded for 4 trials per day. By training day 3, a statistical difference in latency between genotypes emerged (Figure 4A). Moreover, when linear regression was performed on the individual trials for days 2 and 3, the slope of the line corresponding to *Sepp1*(-/-) mice latencies was not significantly different from 0 (slope = 0.5), indicating that during those days of training the animals did not remember the location of the hidden platform (Figure 4B). As expected, the slope of the line corresponding to *Sepp1*(+/+) mice latencies was significantly less than 0 (slope=-3.2), indicating spatial learning of the location of the hidden platform was occurring (Figure 4B). A probe trial was given on day 5 and no difference in the time spent searching the target quadrant was observed between the genotypes (Figure 4C). Training resumed on day 6 and again a difference in escape latency was observed (Figure 4A). To test whether an increased time between training and probe trial would reveal a genotype-dependent difference in long-term memory retention, we trained on day 7, then again on day 10 and performed a probe trial. We found no genotype-dependent difference in the escape latencies (Figure 4A), or in the time spent searching the target quadrant (data not shown). A visible platform test was performed 2 hrs after training on day 10. Latency to find the flagged platform located opposite of training platform was recorded. Both genotypes showed similar latencies to find the new platform (Figure 4D p = 0.70). Taken together, these results suggest that *Sepp1*(-/-) mice have a subtle, yet significant spatial learning deficit that is not the result of impaired visual acuity, swimming ability, or motivation and can be overcome with continued training.

### *Sepp1*(-/-) mice exhibit altered basal synaptic transmission and short-term plasticity in area CA1 of the hippocampus

**Figure 5:**
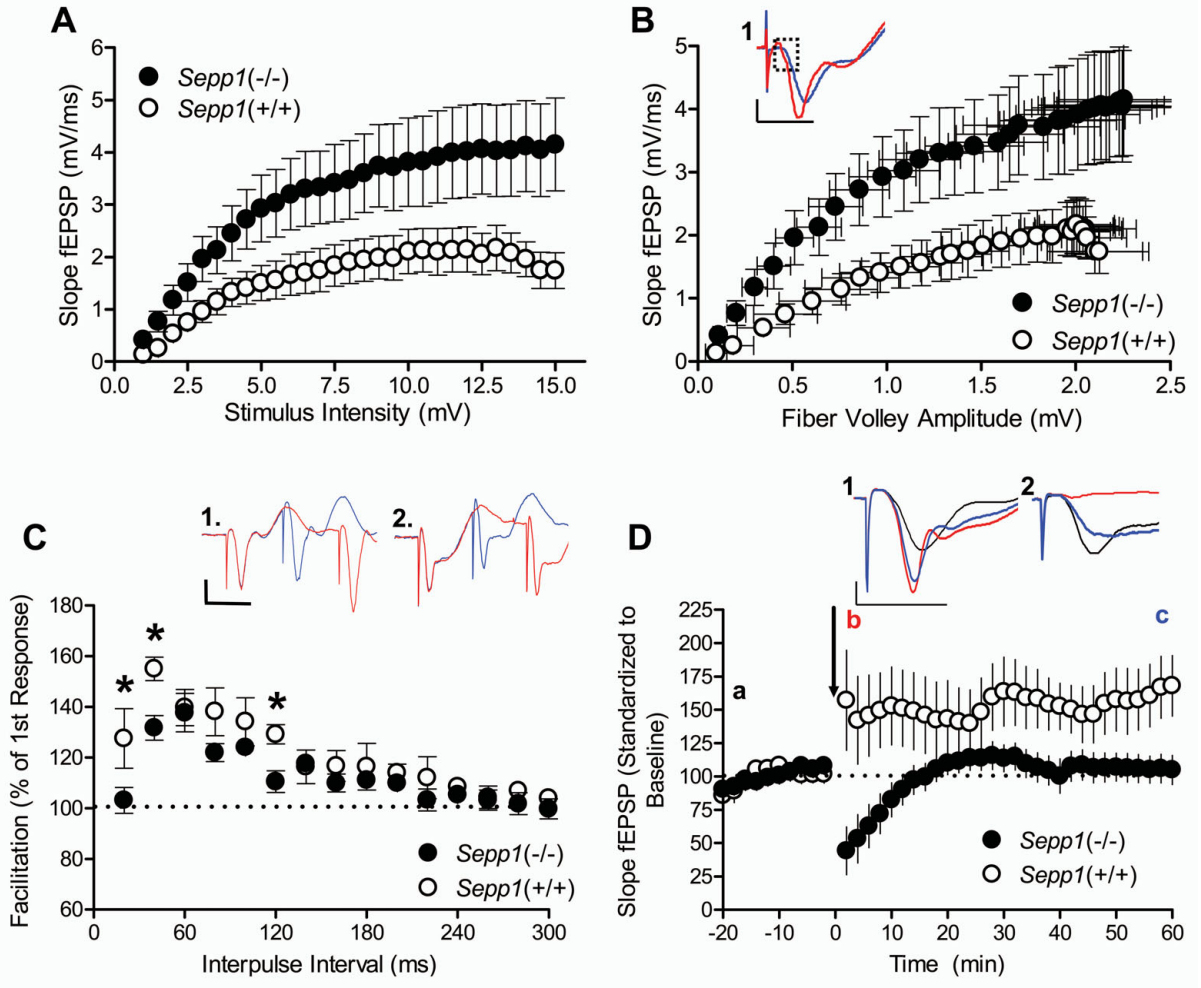
***Sepp1*(-/-) mice exhibit enhanced synaptic transmission and severe synaptic placticity defects**. **(A) **Input-output relationship of the slope of the CA1 field excitatory postsynaptic potential (fEPSP) in response to increasing stimulation of the Schaffer collateral fibers. Slices obtained from *Sepp1*(-/-) mice exhibit greater evoked fEPSP slopes than wild type controls (*Sepp1*(-/-) n = 12, *Sepp1*(+/+) n = 8). Nonlinear regression zero to top analysis confirms that the curves are different (p < 0.0001). **(B) **Relationship between the slope of the evoked fEPSPs from panel A and the corresponding fiber volley amplitude. *Sepp1*(-/-) slices exhibit a greater postsynaptic response than wild type slices to similar presynaptic depolarization. Nonlinear regression zero to top analysis confirms that the curves are different, p < 0.0001) Inset: Representative traces of half the maximum fEPSP slope show a greater fEPSP slope for *Sepp1*(-/-) slices (red) compared to wild type slices (blue) despite similar fiber volley amplitudes (dashed box). **(C) **Percent paired-pulse facilitation (PPF) achieved with increasing inter-pulse intervals. *Sepp1*(-/-) slices have significantly reduced PPF at 20, 40 and 120 ms inter-pulse intervals (*Sepp1*(-/-) n = 12, *Sepp1*(+/+) n = 8; 20 ms *p = 0.04, 40 ms *p = 0.03, 120 ms *p = 0.01). Inset: Representative PPF traces at 20 ms (blue) and 40 ms (red) inter-pulse intervals from (1) *Sepp1*(+/+) slices and (2) *Sepp1*(-/-) slices. **(D) **Long-term potentiation induced by high frequency stimulation (HFS: 100 Hz, 1 sec × 2, 20 sec interval). *Sepp1*(-/-) slices fail to potentiate following HFS (*Sepp1*(-/-) n = 12, *Sepp1*(+/+) n = 8; ANOVA p < 0.0001). Inset: Representative traces from (1) *Sepp1*(+/+) slices and (2) *Sepp1*(-/-) slices at time pints a, b and c (a: black-first baseline recording, b: red-2 min post HFS, c: blue-60 min post HFS). All trace scale bars are 1 mV by 10 ms.

We next sought to determine if the spatial learning deficit observed in *Sepp1*(-/-) mice correlated with a defect in synaptic plasticity. To address this question, we examined the physiologic properties of these mice in area CA1 of the hippocampus, a brain area necessary for normal spatial learning and memory formation. First, we assessed Schaffer collateral-CA1 synaptic connectivity by examining the general input-output of synaptic responses. We stimulated the presynaptic fibers with increasing stimulus intensities and recording the evoked CA1 field excitatory postsynaptic potentials (fEPSPs) in stratum radiatum at Schaffer collateral synapses. As stimulus intensity increased, the *Sepp1*(-/-) slices exhibited significantly greater fEPSP slopes than the wild type slices (Figure 5A), suggesting an increase in afferent activation. To determine if this possibility contributed to the increase in evoked *Sepp1*(-/-) fEPSP amplitude, we analyzed the relationship between the evoked fEPSP slope and the corresponding fiber volley amplitude, which is a measure of presynaptic depolarization. We found that for similar fiber volley amplitudes, the *Sepp1*(-/-) slices exhibited greater fEPSP slopes than slices obtained from the *Sepp1*(+/+) (Figure 5B). This result suggests that *Sepp1*(-/-) animals have enhanced synaptic transmission in area CA1 of the hippocampus.

The enhanced basal synaptic transmission suggests that synapses in *Sepp1*(-/-) mice might have an improved ability to undergo synaptic plasticity. Paired-pulse facilitation (PPF) is a form of short-term plasticity. When two pulses at a short interpulse interval are given to the afferent pathway, the postsynaptic response to the second stimulus is increased when compared with the first response. This phenomenon is understood to be due to residual calcium in the presynaptic terminal that facilitates neurotransmitter release upon the second stimulation resulting in the subsequent increase in the post synaptic response [26]. We tested PPF by delivering paired stimuli at intervals ranging from 20 to 300 ms apart. The *Sepp1*(-/-) slices showed significantly reduced facilitation to the paired-pulse stimulation at intervals of 20, 40 and 120 ms (Figure 5C). Taken together with the input-output relationship data, these results suggest that changes in both presynaptic (altered neurotransmitter release) and postsynaptic properties (enhancement of fEPSP slopes) result from a loss of selenoprotein P.

A type of long-term plasticity exhibited by the Schaffer collateral-CA1 pathway is long-term potentiation (LTP). Two trains of high frequency stimulation (HFS: 100 Hz, 1 sec) spaced 20 s apart faithfully induces LTP in slices obtained from wild type mice characterized by a transient and highly robust post-tetanic increase in the slope of the fEPSP that is followed by a sustained and less robust increase (Figure 5D). Surprisingly, we found that this stimulation paradigm failed to elicit LTP in the *Sepp1*(-/-) slices. Strikingly, not even a modest transient post-tetanic increase was observed. In fact, we observed the opposite; *Sepp1*(-/-) slices responded to the HFS with a robust transient depression of the fEPSP slope (Figure 5D). We performed multiple HFS (2 trains of 100 Hz stimulation, separated by 20 sec repeated 4 times with each pairing given 5 minutes apart) on *Sepp1*(-/-) slices to determine if a stimulation threshold was preventing LTP induction. We found that this HFS protocol did not induce potentiation greater than our standard two train 100 Hz protocol (data not shown). This suggests that the LTP deficit in *Sepp1*(-/-) mice is independent of the amount of HFS presynaptic input, but does not rule out the possibility of occluded LTP.

### *Sepp1*(+/+) mice feed a selenium deficient diet recapitulate the *Sepp*(-/-) phenotype

**Figure 6:**
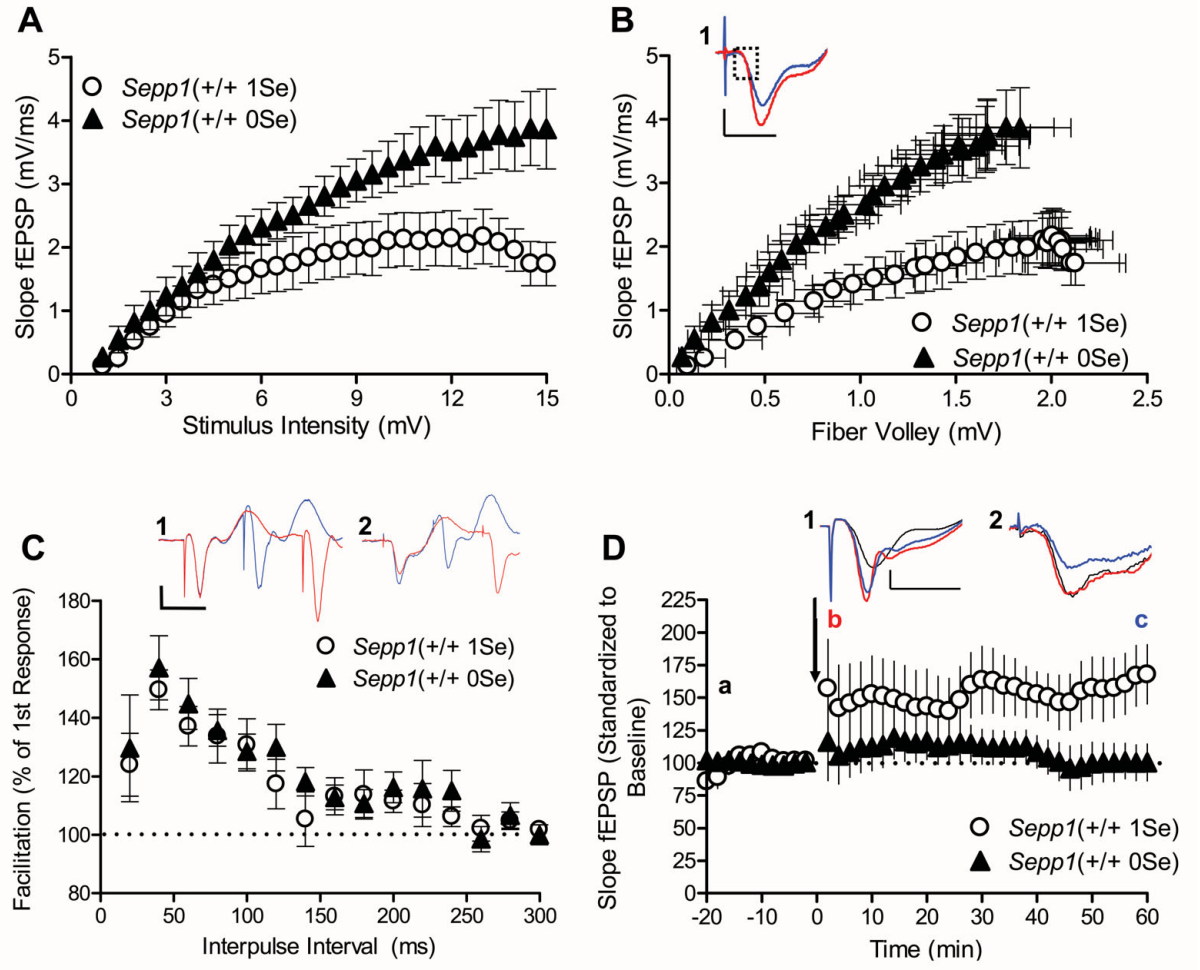
**Adult selenium deficiency enhances synaptic transmission and inhibits long-term potentiation**. **(A) **Input-output relationship of the slope of the CA1 field excitatory postsynaptic potential (fEPSP) in response to increasing stimulation of the Schaffer collateral fibers. Adult selenium deficiency increases evoked fEPSP slopes (*Sepp1*(+/+ 0Se) n = 11, (*Sepp1*(+/+ 1Se) n = 8). Nonlinear regression zero to top analysis confirms that the curves are different (p < 0.0001). **(B) **Relationship between the slope of the evoked fEPSPs from panel A and the corresponding fiber volley amplitude. Adult selenium deficiency increases the postsynaptic response to fixed presynaptic depolarization. Nonlinear regression zero to top analysis confirms that the curves are different, p < 0.0001) Inset: Representative traces of half the maximum fEPSP slope show a greater fEPSP slope for *Sepp1*(+/+ 0Se) slices (red) compared to *Sepp1*(+/+ 1Se) (blue) despite similar fiber volley amplitudes (dashed box). **(C) **Percent paired-pulse facilitation (PPF) achieved with increasing inter-pulse intervals. Adult selenium deficiency does not effect PPF (*Sepp1*(+/+ 0Se) n = 11, *Sepp1*(+/+ 1Se) n = 8). Inset: Representative PPF traces at 20 ms (blue) and 40 ms (red) inter-pulse intervals from (1) *Sepp1*(+/+ 1Se) slices and (2) *Sepp1*(+/+ 0Se) slices. **(D) **Long-term potentiation induced by high frequencystimulation (HFS: 100 Hz, 1 sec × 2, 20 sec interval). *Sepp1*(+/+ 0Se) slices fail to LTP following HFS (*Sepp1*(+/+ 0Se) n = 11, *Sepp1*(+/+ 1Se) n = 8; ANOVA p < 0.0001). Inset: Representative traces from (1) *Sepp1*(+/+ 1Se) slices and (2) *Sepp1*(+/+ 0Se) slices at time pints a, b and c (a: black-first baseline recording, b: red-2 min post HFS, c: blue-60 min post HFS). The same *Sepp1*(+/+ 1Se) from Figure 5 were compared with *Sepp1*(+/+ 0Se). All trace scale bars are 1 mV by 10 ms.

One apparent role of selenoprotein P is to deliver selenium to the CNS. Hill et al (2004) reported that when *Sepp1*(-/-) mice were fed a diet with a normal level of selenium (0.25 mg Se/kg), they had a 43% reduction in selenium levels in the brain [18]. When *Sepp1*(-/-) are fed a selenium-deficient diet (0 mg Se/kg), they exhibit severe neurological abnormalities and typically do not live past 6 weeks of age, whereas their wild type littermates will survive even with approximately 50% reduction in selenium levels [18]. To test whether the observed *Sepp1*(-/-) alterations of synaptic plasticity were due to the deletion of the selenoprotein P gene during development or reduction of selenoprotein P and selenium levels in the adult brain, we examined basal synaptic transmission and synaptic plasticity in *Sepp1*(+/+) mice that were fed a selenium-deficient diet (0 mg Se/kg; designated *Sepp1*(+/+ 0Se)). Importantly, selenium depletion through dietary restriction results in the absence of selenocysteine and subsequent early translation termination at the site of selenocysteine incorporation of all the selenoproteins, including selenoprotein P [27]. Therefore, *Sepp1*(+/+ 0Se) have reduced selenium in the CNS coupled with a decrease of selenoprotein P [16]. Similar to the *Sepp1*(-/-), hippocampal slices obtained from *Sepp1*(+/+ 0Se) mice exhibit enhanced postsynaptic responses to fixed intensities of increasing stimulation when compared to the *Sepp1*(+/+) mice fed 1 mg Se/kg (designated *Sepp1*(+/+ 1Se); Figure 6A). In figure 6B, the Input-Output relationships for the *Sepp1*(+/+ 0Se) show similar presynaptic depolarization to *Sepp1*(+/+ 1Se) because the fiber volley amplitudes were indistinguishable.

The similarity we saw between *Sepp1*(-/-) mice and *Sepp1*(+/+) 0Se mice in enhanced basal synaptic transmission does not extend to pre-synaptic testing by paired-pulse facilitation. Unlike the *Sepp1*(-/-) slices, the *Sepp1*(+/+) slices, regardless of diet, exhibit normal PPF at all interpulse intervals (Figure 6C). Taken together with the previous observation that *Sepp1*(-/-) slices exhibit reduced PPF, these results suggest that the absence of selenoprotein P during development may account for the defect in PPF.

LTP also is altered in slices obtained from *Sepp1*(+/+) 0Se mice. Similar to the *Sepp1*(-/-) slices, *Sepp1*(+/+) 0Se slices do not exhibit LTP in response to HFS. However, unlike the *Sepp1*(-/-) slices, *Sepp1*(+/+) 0Se slices do not respond to HFS with a robust transient depression of the fEPSP slope (Figure 6D), suggesting that the combination of presynaptic and postsynaptic dysfunction in *Sepp1*(-/-) mice may underlie their significant post-tetanic depression. Moreover, this observation paired with the PPF data suggests that a chronic reduction in selenium during adulthood does not recapitulate completely the alterations in synaptic function that result from genetic abolition of selenoprotein P.

## Discussion

The exact role(s) of selenoprotein P in tissues throughout the body are unknown, but since its discovery in 1977 several unique characteristics of the protein have been identified (for review see [28]). First, selenoprotein P incorporates 10 selenocysteine residues in the primary protein [12]. In contrast, other identified selenoproteins exist with the incorporation of a single selenocysteine. Second, selenoprotein P is an abundant plasma protein found to be made in most tissues [13,14]. High concentrations of selenoprotein P mRNA are in the liver, and the liver secretes selenoprotein P into plasma and interstitial fluid [29]. Finally, selenoprotein P contains two distinct protein domains. The first domain consists of the N-terminal domain residue up to just before the second incorporated selenocysteine (AA 1–240). This domain contains a heparin binding motif and exhibits modest peroxidase activity [30-32]. The second domain extends from the second selenocysteine to the remaining protein and contains the other ~nine selenocysteines. Taken together, this suggests that selenoprotein P serves the essential role of transporting necessary selenium to other tissues in the form of selenocysteine, while maintaining a distinct domain that may serve in tissue-specific targeting, in particular the CNS.

Interestingly, dietary Se restriction of *Sepp1*(-/-) mice maintains relatively high concentrations of selenium in the CNS (*Sepp1*(-/-) 86 ± 12 ng Se/g, *Sepp1*(+/+) 99 ± 27 ng Se/g) compared to other tissues that often show 50–90% reduction in Se content (kidney, testis, liver) [16]. Thus, the CNS appears to demonstrate a priority for selenium that goes beyond the capability for selenoprotein P-dependent selenium delivery. Furthermore, Se deficiency induced through dietary restrictions results in disruption of motor function and memory formation [16,25], but these observation may be a result of neuronal cell death in the CNS. Thus, the present studies were performed in order to better define the contribution of selenoprotein P to synaptic function beyond selenium delivery [17].

We find distinct behavioral phenotypic similarities and differences between *Sepp1*(-/-) mice and selenium deficiency. For example, *Sepp1*(-/-) show normal overall activity and no change in anxiety. In contrast, selenium deficiency in mice have reported decreased activity in the open field test and increased anxiety demonstrated as decreased entry to the center of the field [25]. This suggests that cerebellar function is especially sensitive to reduced selenium, which is supported by the deficit in balance and coordination determined by the cerebellum-dependant rotorod test. Spatial learning assessed with the Morris hidden platform test is disrupted in both *Sepp1*(-/-) mice and selenium-deficient mice, although the deficit in *Sepp1*(-/-) mice is subtle and can be overcome with continued training. The increased latency time for days 3, 4 and 6 suggest a learning deficit in acquisition, but the lack of a defect following the probe tests on day 5 suggests that the retention of memories once formed are unaffected. The subtlety of this learning deficit is more apparent in light of normal associative fear conditioned learning in *Sepp1*(-/-) mice compared to *Sepp1*(+/+).

Seeing no abnormal phenotype for the hippocampal-dependant fear conditioning test, we did not expect to see any unusual electrophysiology results. However, the most surprising results of these studies were seen in the electrophysiologic characterization of synaptic function in area CA1 in the hippocampus. *Sepp1*(-/-) mice show a significantly higher output with a given stimulus compared to *Sepp1*(+/+). The increase in the slope of the fEPSP at a given stimulus without a change in the fiber volley amplitude suggests that the defect may reside with postsynaptic function. This is supported by the severe deficit of LTP in *Sepp1*(-/-) mice, which is a predominately postsynaptic-dependent phenomenon. However, decreased PPF at short interpulse intervals is characteristic of presynaptic dysfunction. Interestingly, our results from *Sepp1*(+/+) mice on a 0 mg Se/kg diet show altered synaptic transmission and reduced LTP similar to that of *Sepp1*(-/-) mice. Thus, it is likely that selenoprotein P deficiency has no deleterious developmental consequences, but reductions in selenium, whether through the disruption of selenoprotein P gene or dietary selenium restriction, results in discernable differences in synaptic function. Regardless, it is interesting that these mice exhibit a very severe defect in synaptic plasticity across Schaffer collateral synapses of the hippocampus, yet show only subtle defects in the Morris hidden platform water test and normal associative fear conditioned learning, two distinct hippocampus-dependent behavioral processes.

What might be the mechanism underlying the changes in synaptic function? We can speculate on at least three putative scenarios. First, absence of selenoprotein P may result in diminished anti-oxidant capacity resulting in reduced LTP and disruption of presynaptic responses. However, most research shows that inhibition of antioxidants leads to profound memory disruption as well as disruption of LTP [33,34], a phenotype we don't see in our *Sepp1*(-/-) mice. Second, the reduced selenium content in the CNS may be disrupting the specific function of a selenoprotein involved in synaptic plasticity. Homology studies have identified a number of selenium containing proteins, but none of these are obvious candidates as plasticity proteins. Finally, selenoprotein P may be acting as a signaling molecule through an as yet undetermined receptor. Receptor-dependent endocytosis of selenoprotein P would be a likely mechanism for selenium delivery to cells of the CNS. Numerous signal transduction pathways intimately involved in synaptic plasticity and learning and memory rely on ligand-induced endocytosed receptors [35-37]. Selenoprotein P deficiency, through direct or indirect interactions, may impact an important signaling system.

The present studies establish that selenoprotein P deficiency results in subtle spatial learning deficits and severe synaptic plasticity defects. It is difficult to discern whether this is due to selenoprotein P itself, or the loss of selenium transport to the CNS. However, the use of adult *Sepp1*(+/+) mice maintained on a 0 mg Se/kg diet suggests that the predominance of synaptic dysfunction in *Sepp1*(-/-) mice is not a result of developmental abnormalities. Future studies will help define whether selenoprotein P is acting as a signaling molecule in the CNS or simply facilitates the systemic distribution of necessary selenium.

## Abbreviations

Sepp; Selenoprotein P, *Sepp1*; Selenoprotein P gene, HFS; High Frequency Stimulation, PPF; Paired-pulse facilitation
